# Involvement of UDP-Glucuronosyltransferases and Sulfotransferases in the Excretion and Tissue Distribution of Resveratrol in Mice

**DOI:** 10.3390/nu9121347

**Published:** 2017-12-12

**Authors:** Michaela Böhmdorfer, Akos Szakmary, Robert H. Schiestl, Javier Vaquero, Juliane Riha, Stefan Brenner, Theresia Thalhammer, Thomas Szekeres, Walter Jäger

**Affiliations:** 1 Department of Pharmaceutical Sciences, Division of Clinical Pharmacy and Diagnostics, University of Vienna, Vienna 1010, Austria; michaela.boehmdorfer@univie.ac.at (M.B.); juliane.riha@univie.ac.at (J.R.); stefan.brenner@univie.ac.at (S.B.); 2 Institute of Cancer Research and Comprehensive Cancer Center, Department of Medicine I, Medical University of Vienna, Vienna 1010, Austria; akos.szakmary@vetmeduni.ac.at (A.S.); rschiestl@mednet.ucla.edu (R.H.S.); 3 Department of Pathology and Lab Medicine, UCLA School of Medicine and School of Public Health, University of California at Los Angeles, Los Angeles, CA 90095, USA; 4 Saint-Antoine Research Center, Sorbonne University, Paris 75005, France; javiervr84@hotmail.com; 5 Department of Pathophysiology and Allergy Research, Center of Pathophysiology, Medical University of Vienna, Vienna 1010, Austria; theresia.thalhammer@meduniwien.ac.at; 6 Department of Medical and Chemical Laboratory Diagnostics, Medical University of Vienna, Vienna 1010, Austria; thomas.szekeres@meduniwien.ac.at

**Keywords:** resveratrol, metabolism, mice, Sults, Ugts, tissue distribution

## Abstract

Resveratrol is a naturally occurring polyphenolic compound with various pharmacological activities. It is unknown whether the expression of metabolizing enzymes correlates with resveratrol levels in organs and tissues. Therefore, we investigated the metabolism and tissue distribution of resveratrol in mice and assessed its association with the expression of UDP-glucuronosyltransferase (Ugt) and sulfotransferase (Sult) genes. Plasma, urine, feces, and various organs were analyzed using high-performance liquid chromatography at up to 8 h after intragastric resveratrol administration. The metabolism of resveratrol was pronounced, leading to the formation of resveratrol glucuronides and sulfates. Concentrations of resveratrol and its metabolites were high in the gastrointestinal organs, urine, and feces, but low in the liver and kidneys. In lung, heart, thymus, and brain tissues, parent resveratrol levels exceeded the sulfate and glucuronide concentrations. The formation of resveratrol conjugates correlated with the expression of certain *Ugt* and *Sult* genes. Reverse transcription quantitative PCR (RT-qPCR) analysis revealed high mRNA expression of *Ugt1a1 and Ugt1a6a* in the liver, duodenum, jejunum, ileum, and colon, leading to high concentrations of resveratrol-3-O-glucuronide in these organs. Strong correlations of resveratrol-3-O-sulfate and resveratrol-3-O-4′-O-disulfate formation with *Sult1a1* mRNA expression were also observed, particularly in the liver and colon. In summary, our data revealed organ-specific expression of *Sults and Ugts* in mice that strongly affects resveratrol concentrations; this may also be predictive in humans following oral uptake of dietary resveratrol.

## 1. Introduction

**Figure 1:**
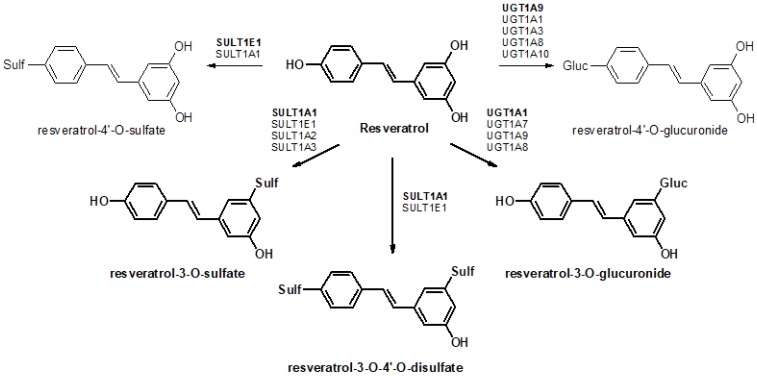
Formation of resveratrol metabolites by various UDP-glucuronosyltransferases (UGTs) and cytosolic sulfotransferases (SULTs) in humans. Conjugates in bold represent the main metabolic pathway.

Resveratrol (3,4′,5-trihydroxy-trans-stilbene) is a naturally occurring compound that is produced by a wide variety of plants, berries, and fruits, and is mainly found in the skin of grapes and in red wine [1]. Interest in resveratrol has increased due to various studies reporting its role in improving the outcomes of various pathologies, e.g., cardiovascular and neurodegenerative diseases, inflammation, viral infections, and cancer [2,3,4]. These beneficial effects on health are observed despite extremely low bioavailability and rapid clearance from the circulation [4,5], based on extensive metabolism in humans and animal models [6,7,8,9]. Previously, data from our laboratory demonstrated that resveratrol is biotransformed in the rat liver into six different products [10], with resveratrol-3-O-glucuronide, resveratrol-3-O-4′-O-disulfate, and resveratrol-3-O-sulfate being the most abundant metabolites. In humans, resveratrol glucuronides are the predominant biotransformation products when low doses of resveratrol are ingested, whereas for higher doses, sulfates are mainly generated [11]. As most resveratrol conjugates have decreased pharmacological activity, first-pass metabolism in the gut and liver has a significant influence on the efficacy of resveratrol. Sulfation and glucuronidation of resveratrol are carried out by UDP-glucuronosyltransferases (UGTs) and cytosolic sulfotransferases (SULTs), respectively, and the expression levels of these enzymes have a major influence on the profile of resveratrol conjugates generated in different organs of the body (Figure 1). Therefore, it is important to determine the tissue-specific expression patterns of these enzymes. Previous investigations have mainly focused on the analysis of resveratrol and its metabolites in plasma, urine, or feces [4,6,12], as well as the distribution in various organs [9,13,14]. To the best of our knowledge, there are no data in the literature regarding the expression of resveratrol-metabolizing enzymes in tissue samples and their correlation with resveratrol concentrations.

Previously, we have shown that resveratrol is selectively metabolized to resveratrol-3-O-sulfate in 12 of 13 human breast cancer tissue specimens [15]. Reverse transcription quantitative PCR (RT-qPCR) analysis of paired samples from control and tumor tissues revealed mRNA expression of SULT1A2, SULT1A3, and SULT1E1, which have all been shown to catalyze resveratrol biotransformation [16]. As SULT1A1 mRNA expression was below the detection limit in all samples and SULT1E1 mRNA was only detectable in a few samples, the cellular localization of SULT1A3 was assessed by indirect immunofluorescence on paraffin-embedded sections from control and malignant breast tissues, clearly indicating a correlation of RT-qPCR data with the protein expression of this enzyme. Other studies identified the UGT1A family, including UGT1A1, UGT1A6, UGT1A7, UGT1A9, and UGT1A10, to be responsible for catalyzing the formation of resveratrol-3-*O*-glucuronide and resveratrol-4′-*O*-glucuronide [17,18].

However, no data are available regarding the association of UGT and SULT mRNA expression with resveratrol glucuronidation and sulfation in tissue samples. Therefore, in the present study, we conducted feeding studies in mice, and time-dependently quantified resveratrol and its main glucuronides and sulfates in plasma and selected tissues by using a sensitive high-performance liquid chromatography (HPLC) assay. Furthermore, the mRNA expression levels of the major isoenzymes responsible for resveratrol metabolism were investigated in order to determine the association between tissue-specific expression and metabolite formation.

## 2. Material and Methods

### 2.1. Chemicals

Resveratrol (3,4′,5-trihydroxy-trans-stilbene) was obtained from Sigma-Aldrich (Munich, Germany). Resveratrol-3-O-glucuronide, resveratrol-3-O-sulfate, and resveratrol-3-O-4′-O-disulfate were obtained from Santa Cruz Biotechnology (Dallas, TX, USA). Methanol and water were of HPLC grade and were purchased from Merck (Darmstadt, Germany). All other chemicals and solvents were commercially available, of analytical grade, and used without further purification.

### 2.2. Animal Experiments

Male wild-type C57BL/6 Oca2^p−un^ (weight, 23–28 g) were obtained from the University of California at Los Angeles (UCLA). Animals were housed in a temperature-, light-, and humidity-controlled environment and fed a SSNIFF™ standard diet and water (pH 3.5) ad libitum. Resveratrol, as a PBS solution containing 1% Tween 20 and 1% ethanol, was administered intragastrically by oral gavage feeding in overnight-fasted mice (aged 1.5–2.5 months) at a single dose of 10 mg/kg resveratrol. Blood samples (300 µL each) were obtained prior to sacrifice of the animals, and were collected into EDTA-coated tubes. For the tissue distribution study, groups of mice (*n* = 3 per group) were sacrificed at 0.5, 1, 1.5, 2, 4, 6, and 8 h post-dosing, respectively. Subsequently, the spleen, liver, kidney, heart, lung, stomach, duodenum, jejunum, ileum, cecum, colon, thymus, muscle, and brain were rapidly excised and washed with NaCl (0.9%) to remove residual blood containing resveratrol and its metabolites. Tissues were wiped with filter paper, weighed, immediately frozen in liquid nitrogen, and maintained at −80 °C until homogenization. For RT-qPCR studies, groups of four mice were used without previous gavage. The experimental protocol was approved by the Committee on the Ethics of the Austrian Ministry of Science (No. 66.009/122-II/10b/2010).

### 2.3. Sample Preparation

Following the addition of 200 μL of methanol to 100 μL of each plasma sample, samples were centrifuged and an 80 μL aliquot of each supernatant was injected into the HPLC column. Mouse tissue samples were thawed on ice at 4 °C, mixed with a threefold amount of 5 mM ammonium acetate/acetic acid buffer (pH 7.4), and then minced using an Ultra-Turrax^®^-homogenizer, four times for 10 s each, on ice. Tissue homogenates were first centrifuged at 13,000× *g* for 10 min, and proteins were eliminated through a second centrifugation by adding methanol (500 µL) to 250 µL of the supernatant. Subsequently, 80 µL of the remaining supernatant was injected onto the HPLC column. In all cases, sample manipulation was performed while avoiding direct light exposure to prevent possible photochemical isomerization of *trans*-resveratrol to the *cis* form.

### 2.4. High-Performance Liquid Chromatography (HPLC) Analysis

Resveratrol and its glucuronidated and sulfated biotransformation products were quantified by HPLC as described previously [19] using a Dionex UltiMate 3000 system (Sunnyvale, CA, USA) equipped with an L-7250 injector, an L-7100 pump, an L-7300 column oven (set at 15 °C), a D-7000 interface, and an L-7400 UV detector (Thermo Fisher Scientific, Waltham, MA, USA) set at a wavelength of 307 nm. Calibration of the chromatogram was accomplished using the external standard method. Linear calibration curves were performed by spiking drug-free cell culture medium with standard solutions of resveratrol, resveratrol-3-*O*-sulfate, resveratrol-3-*O*-4′-*O*-disulfate, and resveratrol-3-*O*-glucuronide to give a concentration range from 0.001 to 10 µg/mL (average correlation coefficients: >0.999). For this method, the lower limits of quantification for resveratrol and resveratrol conjugates were 5 ng and 7 ng, respectively. Coefficients of accuracy and precision for these compounds were <11%.

### 2.5. Reverse Transcription Quantitative PCR (RT-qPCR)

Total RNA was extracted from mouse tissue samples using peqGOLD Trifast™ reagent (peqLAB, Erlangen, Germany), according to the manufacturer’s instructions. The concentration, purity, and integrity of RNA samples were determined by UV absorbance and gel electrophoresis. Total RNA samples (2 µg) were reverse transcribed to cDNA using random hexamer primers and a MultiScribe™ Reverse Transcriptase Kit (ThermoFisher Scientific, Waltham, MA, USA), in accordance with the manufacturer’s instructions. RT-qPCR was performed using RT2 Profiler PCR Arrays (PAMM-069ZA, Qiagen, Hilden, Germany) and RT2 SYBR Green qPCR Master Mix (Qiagen, Hilden, Germany) on 96-well plates in an ABI Prism^®^ 7900HT Sequence Detection System (Thermo Fisher Scientific, Waltham, MA, USA) to analyze mouse phase II enzymes involved in drug metabolism. In order to determine appropriate reference genes, 5 different mouse housekeeping genes were included in the arrays, and Hprt (validated with the Mm01545399TaqMan^®^ Gene Expression Assays, ThermoFisher Scientific, Waltham, MA, USA) was selected as the most appropriate reference gene. Gene expression levels were quantified relative to the values obtained for Hprt. Data analyses were done using the web-based analysis software from Quiagen.

### 2.6. Data Analysis

For the quantifications of resveratrol and its metabolites in plasma, urine, feces, and various organs, a group of three mice was used. The area under the concentration curves from 0 to 8 h (AUCs_0–8h_) was calculated using the linear trapezoidal rule with the program Phoenic WinNonlin version 1.5 (Centera Inc., Princeton, NJ, USA). The maximal plasma, urine, feces, and tissue concentration (C_max_) and the time after administration of resveratrol when C_max_ was reached (T_max_) were estimated from Table 1, Table 2, Table 3 and Table 4. All concentrations and the values for C_max_ and AUC were expressed as mean ± SD calculated using the GraphPad Prism 6.0 (GraphPad, San Diego, CA, USA).

## 3. Results

### 3.1. Tissue Levels of Resveratrol and Its Conjugated Metabolites

Mice received intragastric resveratrol (10 mg/kg), and the levels of resveratrol and its main metabolites (resveratrol-3-O-sulfate, resveratrol-3-O-4′-O-disulfate, and resveratrol-3-O-glucuronide) were measured in the plasma, urine, and feces and in gastrointestinal organs (stomach, duodenum, jejunum, ileum, colon), the immune system (spleen), and the liver, kidney, heart, lung, thymus, muscle, and brain, respectively. Table 1, Table 2, Table 3 and Table 4 summarize the concentrations of resveratrol and its metabolites in mouse tissues collected at 0.5, 1, 1.5, 2, 4, 6, and 8 h after intragastric resveratrol administration. The results indicate that resveratrol and its metabolites were rapidly and widely distributed in the body.

In all tissues, except the spleen, kidney, ileum, cecum, colon, and thymus, resveratrol levels peaked at 30 min. The highest levels were observed for the cecum (163.5 nmol/g) after 1.5 h, and the stomach (128.9 nmol/g) after 30 min, exceeding the levels in the liver and brain by up to 300-fold. Peak values in the ileum and kidneys were reached after 1 h.

Similar observations were made for the glucuronidated and sulfated resveratrol metabolites. Resveratrol-3-O-glucuronide levels were highest in the stomach and duodenum after 30 min, and were even higher in the ileum after 1 h, at levels 30- to 50-fold higher than those in the spleen and muscle. The concentration of resveratrol-3-O-sulfate was also highest in the stomach and duodenum after 30 min (96.2 nmol/g), and in the ileum after 1 h (178.2 nmol/g).

As shown in Table 4, resveratrol-3-O-4’-O-disulfate concentrations were highest in the stomach and duodenum after 30 min and in the ileum after 1 h, reflecting the patterns exhibited by resveratrol and the two aforementioned metabolites. Interestingly, all glucuronidated and sulfated metabolites were undetectable in brain tissues.

Resveratrol levels in the plasma were low and peaked at 30 min. The same was true for the metabolites; an approximately threefold-higher concentration was observed in the plasma for resveratrol-3-O-glucuronide and resveratrol-3-O-sulfate compared with resveratrol 3-O-4’-O-disulfate. Contrary to in the plasma, resveratrol and its conjugates were found at high concentrations in the feces; resveratrol levels in the urine were negligible.

Table 5 shows the area under the time curves (AUC) of plasmatic, urinary, fecal, and tissue concentrations of resveratrol and its metabolites for the 8 h immediately following resveratrol administration. The highest resveratrol-3-O-glucuronide concentrations were predominantly detected in the urine. The most obvious change in resveratrol and its metabolite concentrations occurred in the spleen, in which the AUC for resveratrol was 99.7 times higher than that for resveratrol-3-O-sulfate. In general, the amount of resveratrol-3-O-4′-O-disulfate formed was low compared with the two other metabolites, and was below the detection limit in the spleen, thymus, and brain. However, in the ileum and urine, the AUCs of resveratrol-3-O-4′-O-disulfate were 1.6 and 22 times higher, respectively, than that for free resveratrol.

**Figure 2:**
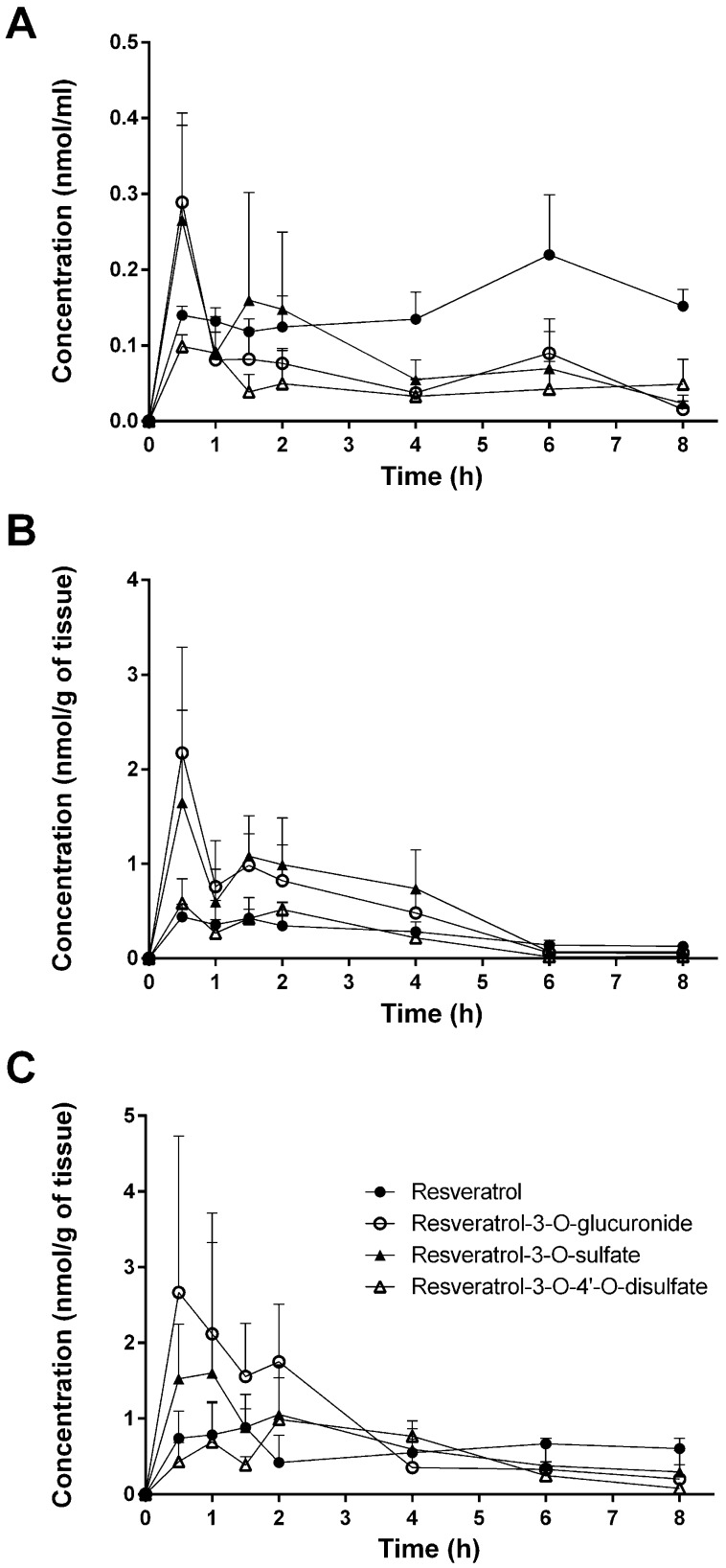
Concentrations of resveratrol and its metabolites in plasma (**A**) and in liver (**B**) and kidney (**C**) tissues after a single intragastric gavage of 10 mg/kg of resveratrol in mice. Values are presented as the mean ± SD; *n* = 3.

**Figure 3:**
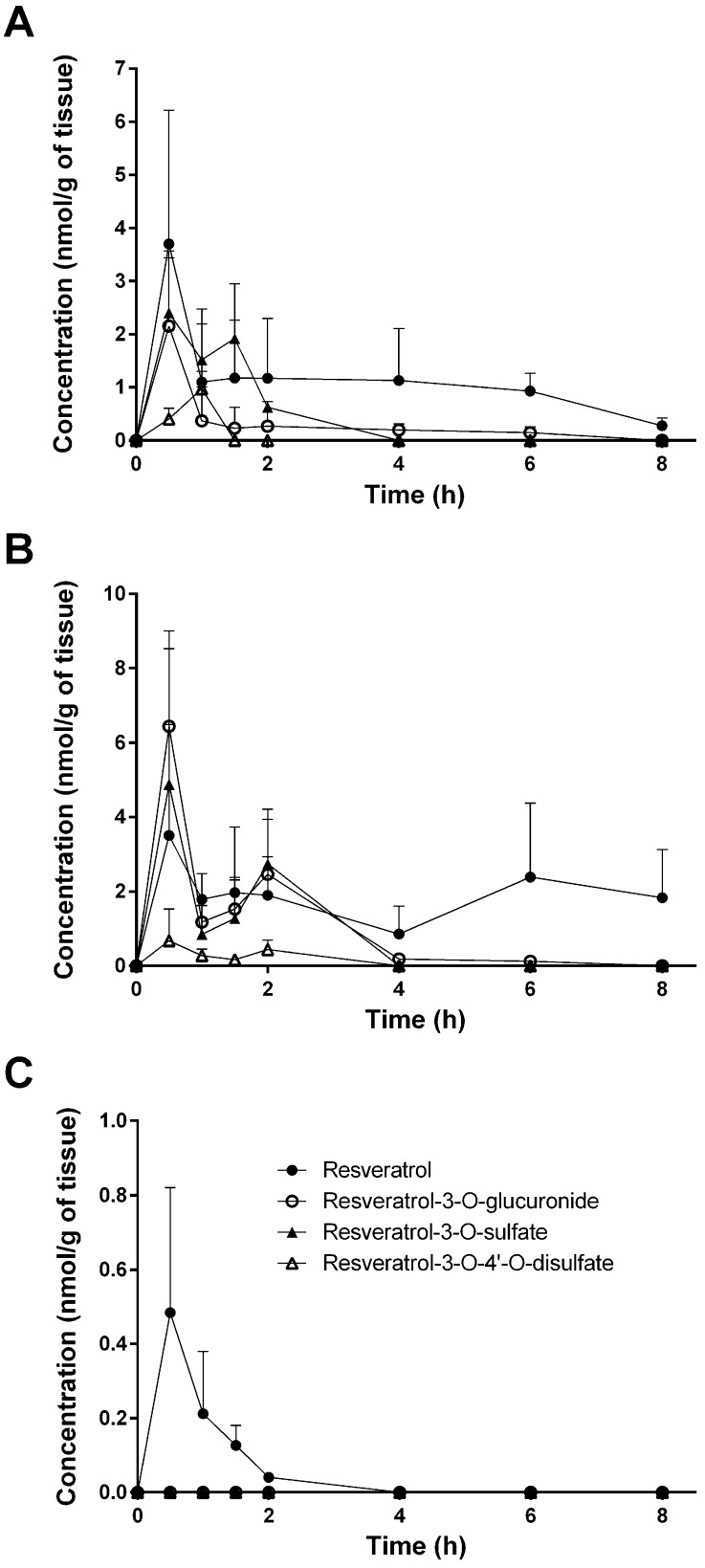
Tissue concentrations of resveratrol and its metabolites in heart (**A**), lung (**B**), and brain (**C**) after a single intragastric gavage of 10 mg/kg of resveratrol in mice. Values are presented as the mean ± SD; *n* = 3.

Figure 2 and Figure 3 provide an overview of resveratrol and its three main metabolites in the plasma, liver, kidney, heart, lung, and brain during the time course of 8 h. As shown in Figure 2A, parent resveratrol and resveratrol-3-O-glucuronide peaked in the plasma at 30 min, with a second peak at 6 h, which indicates enterohepatic recirculation. In the liver and kidneys (Figure 2A,B), resveratrol and its conjugates decreased and were markedly low after 6 h, particularly in the liver. Differences were also observed in the distribution of resveratrol in the heart and lungs, where resveratrol concentrations only slightly decreased, or even increased, after 6 h (Figure 3A,B). As shown in Figure 3C and in Table 2, Table 3 and Table 4, parent resveratrol was found in the brain (but its metabolites were not), with a maximal concentration after 30 min, followed by a rapid decline.

**Figure 4:**
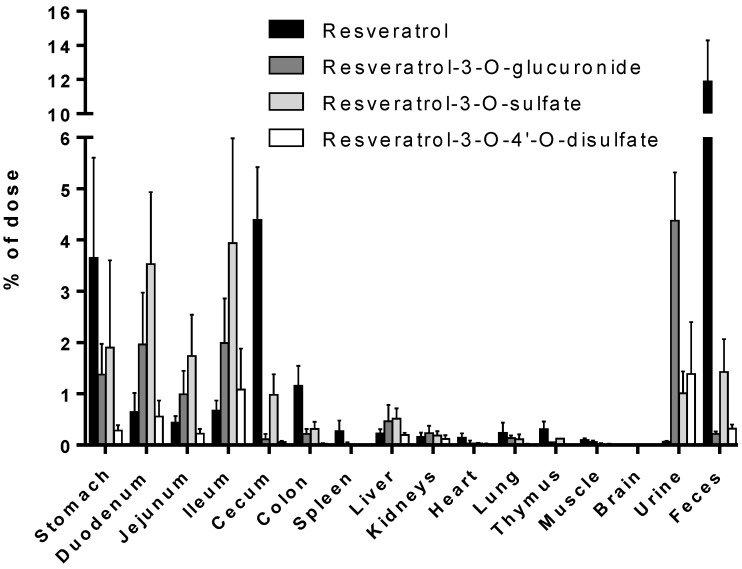
Recovery of resveratrol and its metabolites in mice tissues after a single gastric gavage (0–8 h) as a percentage of the total amount administered. Values are presented as the mean ± SD; *n* = 3.

Figure 4 gives an overview of the total amount (calculated as the percentage of the applied dose) of resveratrol and its metabolites in mouse tissue samples, plasma, urine, and feces during the time of the experiment (0–8 h). We quantified 21.3% of the metabolites in the gastrointestinal tract, of which the most abundant metabolite was resveratrol-3-O-sulfate (12.4% of the initial resveratrol amount administered). Interestingly, ~11% of the unconjugated resveratrol was detected in the stomach, duodenum, jejunum, cecum, and colon. The recovery of resveratrol, resveratrol-3-O-sulfate, resveratrol-3-O-4′-O-disulfate, and resveratrol-3-*O*-glucuronide was 0.7, 1.0, 1.4, and 4.4% in the urine, and 11.9, 1.4, 0.3, and 0.2% in the feces, respectively. Only ~2% of the dose was recovered as resveratrol-3-O-glucuronide or resveratrol-3-*O*-sulfate from all organs analyzed, whereas approximately 1.5% was quantified as unconjugated resveratrol.

### 3.2. Expression of Sult and Ugt Genes in Mouse Tissues and Organs

**Figure 5:**
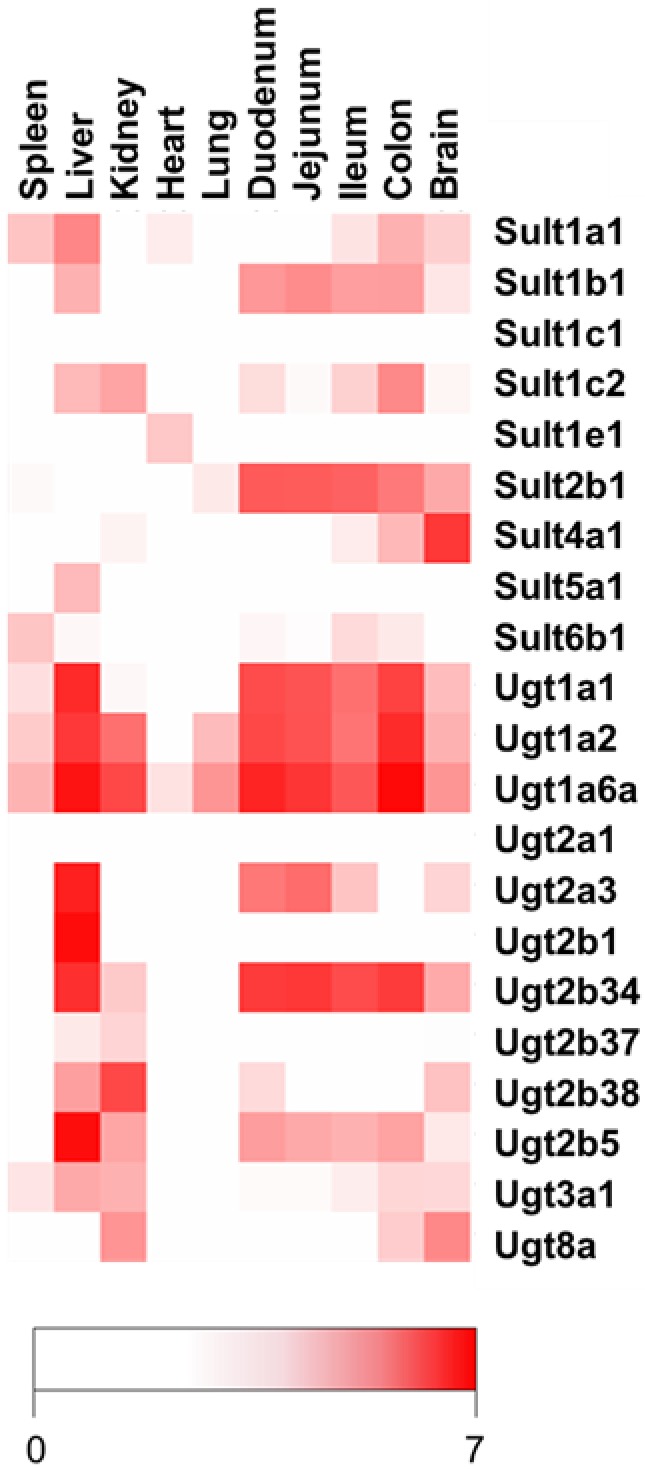
Expression map showing mRNA expression of various Sults and Ugts in tissue samples from mice. The image map (Java TreeView [23]) shows the pattern of enzyme expression as determined by reverse transcription quantitative PCR relative to the expression of Hprt (selected as the most appropriate reference gene).

To investigate the expression of enzyme systems that may be involved in the phase II metabolism of resveratrol, RT-qPCR analyses were performed for 9 Sult and 12 Ugt genes in selected mouse organs. In accordance with previous data on the differences in Ugt and Sult expression in mouse organs [20,21,22], we identified organ-specific expression of different isoforms (see Figure 5). From the Ugt family 1, *Ugt1a1*, *Ugt1a2*, *Ugt1a6a*, and *Ugt2b34* mRNAs were highly expressed in the liver and intestines (duodenum, jejunum, ileum, and colon). *Ugt2a3*, a member of the Ugt family 2, was expressed in the liver, duodenum, and jejunum, but was negligible in the ileum and not detected the colon. *Ugt2b38* and *Ugt2b5* were observed at high levels in the liver and spleen, respectively, and at considerably lower levels in the duodenum and brain, while *Ugt2b1* mRNA expression appeared to be liver-specific. *Ugt8a* mRNA expression was high in the liver and brain, whereas *Ugt3a1* was expressed at only low levels in the liver, spleen, colon, and brain. *Ugt2a1* mRNA expression in all tissue samples was below the detection limit, and a very low level of *Ugt2b37* expression was detectable only in the liver and spleen.

The expression of the *Sult1a1* gene, the mouse homolog of human SULT1A1, was highest in the liver, followed by the colon, spleen, and brain. *Sult1b1* and *Sult2b1* mRNAs were primarily expressed in the intestines (duodenum, jejunum, ileum, and colon), while *Sult1c2* mRNA expression was highest in the colon, followed by the kidney. Interestingly, *Sult4a1*, *Sult5a1*, and *Sult6B1* mRNAs exhibited selective expression in the brain, liver, and spleen, respectively. *Sult1c1* and *Sult6b1* mRNA transcripts were below the detection limit in all investigated tissue samples.

## 4. Discussion

In the present study, tissue distribution of resveratrol and its three major metabolites (resveratrol-3-O-sulfate, resveratrol-3-O-4′-O-disulfate, and resveratrol-3-O-glucuronide) in the plasma, urine, feces, and tissues of mice was investigated in male mice after oral administration of resveratrol (10 mg/kg).

A single dose of 10 mg/kg was chosen based on the daily intake of resveratrol as dietary supplement (100–500 mg/day) to humans. Furthermore, data from our lab also showed that higher resveratrol concentrations inhibit the formation of resveratrol-3-O-sulfate, which might also be observed in mice [24]. We decided to use male and not female mice in all experiments as recent papers [25,26] described significant gender-dependent differences in the glucuronidation of drugs and flavonoids in mice, with a higher formation rate of glucuonides in females than in males, which might be also true for resveratrol. In humans, such pronounced gender-difference in glucuronidation has not been observed so far.

Despite the numerous studies that have investigated the plasmatic levels of resveratrol and its metabolites in mice, very few have attempted to assess the tissue distribution of this natural compound. Therefore, to the best of our knowledge, the present study constitutes the first report of the time-dependent distribution of resveratrol and its major metabolites in 14 tissues as well as in feces and urine in mice following intragastric administration. No data about the concentration of resveratrol and its conjugates in the spleen, thymus, and muscle have been published so far. Furthermore, we also performed, for the first time, a comparison of concentrations with the presence of phase II metabolizing enzymes in appropriate tissues and organs. The liver, kidneys, and gastrointestinal tract are organs with a high phase II metabolizing activity [27]. After oral administration of resveratrol, our experiments showed that resveratrol was detected predominantly in its glucuronic-acid- and sulfate-conjugated forms in these organs (Figure 4). High levels of resveratrol conjugates could be detected already in the stomach by resveratrol-metabolizing gastric cells. The lower concentration of the three conjugates in the cecum and colon might be due to a decreased expression of the efflux transporters multidrug resistance protein 2 (MRP2) and breast cancer resistance protein (BCRP), as both membrane proteins are substrates for resveratrol and its conjugates [28,29]. Both transporters are able to pump resveratrol glucuronides and sulfates back into the lumen as previously shown in rats [30]. Interestingly, the overall concentration of resveratrol was higher than that of its metabolites in the stomach, cecum, colon, spleen, heart, lung, thymus, and muscle, as well as in the fecal samples (Figure 4). A recent paper of Menet et al. [31] confirmed our results also showing higher resveratrol concentration in mouse heart tissue. This is in contrast to other authors who found higher levels of conjugates in the hearts of rats and pigs [9,13].

The same is true for the lung, for which Juan et al. [32] also showed higher resveratrol concentrations, whereas Lin et al. [13] could only detect conjugates. Interestingly, in the lung, we could only quantify resveratrol at low levels; the concentration of all three metabolites was below the detection limit. The observed poor accumulation of resveratrol in the brain might be explained by the presence of MRP2 and BCRP in the blood–brain barrier, preventing a pronounced uptake into the brain. No detectable resveratrol conjugates in the brain are in line with findings in pigs shown after intragastric administration of 6.5 mg/kg of resveratrol [9]. Other authors, however, could not detect resveratrol and its conjugates in rat and mouse brains [13] or found up to six-fold higher resveratrol-3-*O*-sulfate concentration compared with that of native resveratrol [33]. This discrepancy might be explained by altered doses or different sample preparation steps prior to analysis.

As mentioned above, higher resveratrol concentrations might inhibit resveratrol-3-O-sulfate formation and also change the pattern of metabolites. Indeed, a single dose of 150 mg/kg to mice resulted in up to 5.8-fold higher resveratrol-3-O-glucuronide concentrations in mouse hearts 30 min and 60 min after dosing [31], whereas our data showed slightly higher concentrations of resveratrol-3-O-sulfate levels at the same time points.

Interestingly, tissue, feces, and urine levels of resveratrol and its conjugates did not show the expected decline with time after dosing. This might be explained either with a delayed transport of resveratrol from the stomach to the colon or by a redistribution between organs. This would explain the high concentration in the thymus for free resveratrol after 8 h. A second peak in the plasma after 6 h for unconjugated resveratrol is possibly caused by enterohepatic recirculation of free resveratrol cleaved by β-glucuronidase from resveratrol 3-O-glucuronide after biliary excretion. The maximum concentration of the final metabolic product resveratrol-3-O-4′-O-sulfate in the urine after 6 h, however, can be explained by its longer formation time starting from resveratrol via resveratrol-3-O-sulfate and resveratrol-4′-O-sulfate.

In humans, resveratrol glucuronidation is mainly catalyzed by UGT1A1 and UGT1A9, and, to a minor extent, by UGT1A6, UGT1A7, and UGT1A10 [18,34], whereas sulfation is performed by SULT1A1, SULT1A2, SULT1A3, and SULT1E1 [15,16]. Although there are no data available in the literature regarding the conjugation of resveratrol glucuronides and sulfates by mouse-specific Ugts and Sults, several studies have demonstrated high homology between mouse and human isoenzymes, at least for Ugt1a1, Ugt1a6a, and Sult1A1, with a similarity in the amino acid sequence of 75–90% [35,36], which suggests comparable enzymatic activity for resveratrol.

In the present study, the formation of resveratrol glucuronides and sulfates correlated with the expression of certain *Ugts* and *Sults*. RT-qPCR analysis revealed high mRNA expression of *Ugt1a1* and *Ugt1a6a* in the liver, duodenum, jejunum, and ileum, leading to high concentrations of resveratrol-3-O-glucuronide in these organs. High expression of *Ugt1a1* and *Ugt1a6a* in the liver and small intestines of mice is in accordance with previously reported data [20,22]. A good correlation of resveratrol-3-O-sulfate and resveratrol-3-O-4′-O-disulfate formation with *Sult1a1* mRNA expression was also observed, particularly in the liver and colon.

*Ugt1a2* and *Ugt2b34* were highly expressed in the liver, duodenum, jejunum, and ileum, whereas Sult4a1 mRNA was almost exclusively detected in the brain. Whether Sult2b1, Ugt1a2, and Ugt2b34 also catalyze resveratrol sulfation and glucuronidation is not yet known. However, based on their high expression in the liver and gastrointestinal tract, we assume the involvement of these isoforms in the metabolism of resveratrol in mouse organs. Our data are in line with previous reports also demonstrating high mRNA expression of *Ugt2b5* and *Ugt2b1* in the liver and of *Ugt2b38* in the kidney [36]. Contrary to a study by Saiki et al., which reported the expression of Sult1b1 mRNA exclusively in the liver, the present findings revealed *Sult1b1* mRNA expression in the small and large intestines in mice [37]. This discrepancy may be explained by the fact that the authors used a Northern blot analysis and not the more sensitive RT-qPCR. RT-qPCR analysis may therefore be predictive for the metabolism in tissue samples. Low or no detectable expression of Ugts and Sults, as observed in the spleen, heart, and lung, consequently led to an overall far higher percentage of resveratrol compared with those of its conjugates.

The different distribution of resveratrol and its conjugates in various tissues may be predictive for the observed pharmacological activities of resveratrol. In organs like the spleen, heart, lung, muscle, and brain, resveratrol concentrations might be far too low for any significant biological effect. Although resveratrol glucuronides and sulfates are less active than resveratrol, they may act as a depot, easily converted back to resveratrol by ubiquitously existing human β-glucuronidase and sulfatases. The expression of UGTs and SULTs in the tissues therefore helps to predict the overall pharmacological activity of resveratrol.

Another option to improve oral bioavailibity of resveratrol and, consequently, its efficacy is the inhibition of the glucuronidation in the intestine by UGT-inhibitors. Co-administration of the UGT inhibitor piperin increased the AUC and C_max_ of resveratrol in rat plasma by 229% and 1544%, respectively [38]. This pronounced effect was also observed when resveratrol was administered by a nanoemulsion composed of soybean oil, soy lecithin, and the UGT inhibitor labrasol, again increasing the C_max_ and bioavailability approximately 11- and 5-fold, respectively [39]. These strategies might help to increase concentrations in organs for which we show minor accumulation of resveratrol.

## 5. Conclusions

In conclusion, the present study demonstrated organ-specific metabolization of resveratrol into its major conjugates—resveratrol-3-O-glucuronide, resveratrol-3-O-sulfate, and resveratrol-3-O-4′-O-disulfate—in mice. Metabolite concentration correlated with the distribution of Sult and Ugt genes, which may be predictive in humans after the dietary administration of resveratrol. Therefore, further in vivo studies should focus on the concentration of resveratrol and its metabolites in target tissues, and also take into account the expression levels of enzymes involved in resveratrol conjugation.

## Figures and Tables

**Table 1:** Distribution of unmetabolized resveratrol in plasma (nmol/mL), various organs (nmol/g of tissue), urine (nmol/mL), and feces (nmol/g wet content) of mice at different time-points following the intragastric administration of a single dose of 10 mg/kg resveratrol. Maximum concentrations (C_max_ values) are in bold.

Sample	Resveratrol Concentration						
	0.5 h	1 h	1.5 h	2 h	4 h	6 h	8 h
**Plasma**	**0.140 ± 0.012**	0.132 ± 0.017	0.118 ± 0.017	0.124 ± 0.041	0.135 ± 0.036	0.220 ± 0.079	0.152 ± 0.023
**Spleen**	4.347 ± 3.686	3.434 ± 1.946	3.305 ± 3.473	**7.232 ± 1.050**	3.218 ± 2.983	3.134 ± 1.824	2.229 ± 2.045
**Liver**	**0.439 ± 0.133**	0.358 ± 0.255	0.424 ± 0.217	0.345 ± 0.252	0.281 ± 0.105	0.141 ± 0.023	0.128 ± 0.033
**Kidney**	0.740 ± 0.361	**0.786 ± 0.441**	0.881 ± 0.436	0.418 ± 0.365	0.548 ± 0.140	0.667 ± 0.039	0.602 ± 0.137
**Heart**	**3.698 ± 2.519**	1.097 ± 1.094	1.176 ± 1.091	1.169 ± 1.126	1.129 ± 0.978	0.931 ± 0.339	0.276 ± 0.147
**Lung**	**3.505 ± 2.985**	1.791 ± 0.699	1.968 ± 1.769	1.899 ± 2.056	0.857 ± 0.749	2.388 ± 1.989	1.827 ± 1.300
**Stomach**	**128.9 ± 74.38**	111.8 ± 52.42	71.18 ± 60.49	40.93 ± 35.12	5.065 ± 2.220	1.709 ± 1.490	2.600 ± 1.072
**Duodenum**	**10.01 ± 4.763**	4.505 ± 2.787	6.551 ± 4.581	0.984 ± 0.081	0.752 ± 0.210	1.651 ± 0.673	1.100 ± 0.452
**Jejunum**	**3.590 ± 1.692**	1.693 ± 0.854	2.386 ± 2.288	0.889 ± 0.261	0.485 ± 0.133	0.715 ± 0.044	0.985 ± 0.515
**Ileum**	8.435 ± 2.611	**26.17 ± 19.12**	6.411 ± 2.166	2.074 ± 1.421	1.513 ± 0.512	1.289 ± 1.120	2.428 ± 1.751
**Cecum**	22.66 ± 15.47	11.52 ± 4.843	**163.5 ± 138.5**	30.72 ± 16.22	29.05 ± 19.92	1.761 ± 1.569	2.587 ± 2.235
**Colon**	7.422 ± 2.339	2.292 ± 1.390	**22.04 ± 10.64**	10.34 ± 6.731	9.754 ± 5.745	0.862 ± 1.087	2.655 ± 0.496
**Thymus**	15.01 ± 8.971	**15.21 ± 6.934**	4.784 ± 1.286	2.857 ± 0.192	0.818 ± 0.314	2.351 ± 1.051	11.01 ± 4.994
**Muscle**	**1.530 ± 0.824**	0.680 ± 0.619	0.557 ± 0.489	0.695 ± 1.203	0.555 ± 0.483	0.163 ± 0.128	0.333 ± 0.177
**Brain**	**0.484 ± 0.337**	0.212 ± 0.167	0.127 ± 0.054	0.041 ± 0.014	ND	ND	ND
**Urine**	4.716 ± 0.057	3.510 ± 0.513	**15.70 ± 11.85**	0.779 ± 0.106	5.091 ± 3.238	5.455 ± 2.562	4.472 ± 2.398
**Feces**	49.62 ± 29.79	186.5 ± 135.1	171.8 ± 79.23	292.5 ± 156.6	**336.9 ± 219.2**	22.24 ± 14.37	15.58 ± 13.93

Data are expressed as the mean ± SD; *n* = 3. ND: not detected.

**Table 2:** Distribution of resveratrol-3-O-glucuronide in plasma (nmol/mL), various organs (nmol/g of tissue), urine (nmol/mL), and feces (nmol/g wet content) in mice at different time-points following the intragastric administration of resveratrol at a single dose of 10 mg/kg. Maximum concentrations (C_max_ values) are in bold.

Sample	Resveratrol-3-O-Glucuronide Concentration						
	0.5 h	1 h	1.5 h	2 h	4 h	6 h	8 h
**Plasma**	**0.289 ± 0.102**	0.081 ± 0.055	0.082 ± 0.040	0.076 ± 0.021	0.037 ± 0.019	0.090 ± 0.045	0.016 ± 0.011
**Spleen**	0.785 ± 1.360	**1.908 ± 1.305**	1.767 ± 1.061	ND	ND	ND	ND
**Liver**	**2.173 ± 1.120**	0.758 ± 0.488	0.984 ± 0.333	0.821 ± 0.380	0.480 ± 0.201	0.059 ± 0.101	0.057 ± 0.056
**Kidney**	**2.665 ± 2.066**	2.119 ± 1.598	1.556 ± 0.702	1.749 ± 0.759	0.351 ± 0.377	0.329 ± 0.048	0.202 ± 0.190
**Heart**	**2.155 ± 1.284**	0.370 ± 0.640	0.230 ± 0.398	0.269 ± 0.467	0.198 ± 0.114	0.145 ± 0.112	ND
**Lung**	**6.440 ± 2.566**	1.179 ± 0.658	1.527 ± 0.781	2.459 ± 0.471	0.185 ± 0.120	0.124 ± 0.095	ND
**Stomach**	**72.69 ± 41.68**	35.83 ± 10.97	21.93 ± 7.397	8.483 ± 3.440	2.754 ± 1.625	0.898 ± 0.786	1.140 ± 1.396
**Duodenum**	**46.03 ± 36.63**	15.96 ± 11.51	26.49 ± 13.38	3.761 ± 0.734	1.553 ± 1.101	0.672 ± 0.599	0.445 ± 0.241
**Jejunum**	**11.30 ± 7.849**	3.658 ± 2.922	10.74 ± 2.962	2.610 ± 2.396	0.946 ± 0.508	0.291 ± 0.234	0.183 ± 0.060
**Ileum**	35.07 ± 7.835	**118.6 ± 49.70**	12.26 ± 0.822	3.282 ± 2.030	1.716 ± 0.814	0.492 ± 0.440	0.594 ± 0.328
**Cecum**	1.528 ± 1.299	0.702 ± 0.653	0.627 ± 1.085	**3.167 ± 2.962**	ND	ND	ND
**Colon**	1.845 ± 0.530	0.707 ± 0.540	**4.385 ± 5.582**	3.830 ± 3.202	0.805 ± 0.394	ND	ND
**Thymus**	**12.39 ± 2.539**	ND	ND	ND	ND	ND	ND
**Muscle**	**0.683 ± 0.673**	0.642 ± 0.090	0.622 ± 0.299	0.342 ± 0.309	0.232 ± 0.101	ND	ND
**Brain**	ND	ND	ND	ND	ND	ND	ND
**Urine**	362.7 ± 86.98	**522.5 ± 289.7**	440.1 ± 146.9	147.5 ± 78.32	168.1 ± 118.9	120.5 ± 51.62	96.92 ± 36.83
**Feces**	**22.60 ± 9.050**	ND	ND	ND	ND	1.524 ± 1.321	9.743 ± 1.216

Data are expressed as the mean ± SD; *n* = 3. ND: not detected.

**Table 3:** Distribution of resveratrol-3-O-sulfate in plasma (nmol/mL), various organs (nmol/g of tissue), urine (nmol/mL), and feces (nmol/g wet content) in mice at different time-points following the intragastric administration of resveratrol at a single dose of 10 mg/kg. Maximum concentrations (C_max_ values) are in bold.

Sample	Resveratrol-3-O-Sulfate Concentration						
	0.5 h	1 h	1.5 h	2 h	4 h	6 h	8 h
**Plasma**	**0.265 ± 0.141**	0.089 ± 0.049	0.160 ± 0.142	0.148 ± 0.102	0.055 ± 0.026	0.069 ± 0.049	0.023 ± 0.011
**Spleen**	**0.558 ± 0.966**	ND	ND	ND	ND	ND	ND
**Liver**	**1.649 ± 0.975**	0.596 ± 0.347	1.081 ± 0.428	0.991 ± 0.493	0.739 ± 0.409	0.070 ± 0.122	0.070 ± 0.068
**Kidney**	1.525 ± 0.724	**1.602 ± 1.726**	0.885 ± 0.244	1.055 ± 0.489	0.594 ± 0.374	0.378 ± 0.366	0.295 ± 0.282
**Heart**	**2.405 ± 1.166**	1.517 ± 0.954	1.922 ± 1.031	0.627 ± 0.085	ND	ND	ND
**Lung**	**4.877 ± 3.653**	0.848 ± 0.771	1.271 ± 1.105	2.741 ± 1.465	ND	ND	ND
**Stomach**	**96.16 ± 38.23**	45.50 ± 21.81	36.51 ± 30.13	7.719 ± 4.434	3.605 ± 1.059	3.043 ± 1.057	4.332 ± 0.968
**Duodenum**	**67.25 ± 27.95**	27.08 ± 11.75	59.21 ± 34.83	8.202 ± 2.905	3.141 ± 1.562	1.358 ± 1.179	0.910 ± 0.868
**Jejunum**	13.24 ± 7.097	6.847 ± 4.393	**16.05 ± 12.51**	6.451 ± 3.212	2.370 ± 1.228	0.828 ± 0.637	0.497 ± 0.275
**Ileum**	73.23 ± 19.10	**178.2 ± 125.5**	41.65 ± 15.54	13.48 ± 8.766	6.577 ± 4.057	2.198 ± 0.852	1.583 ± 1.495
**Cecum**	12.67 ± 3.839	2.883 ± 2.691	**24.94 ± 13.31**	12.58 ± 8.254	3.829 ± 1.946	0.590 ± 0.122	0.123 ± 0.213
**Colon**	2.189 ± 0.749	0.929 ± 0.603	**6.117 ± 2.114**	5.133 ± 3.381	1.646 ± 1.982	ND	ND
**Thymus**	**28.31 ± 14.53**	ND	ND	ND	ND	ND	ND
**Muscle**	0.209 ± 0.363	**0.217 ± 0.162**	0.196 ± 0.339	ND	ND	ND	ND
**Brain**	ND	ND	ND	ND	ND	ND	ND
**Urine**	**128.3 ± 33.15**	108.5 ± 51.50	62.23 ± 15.03	54.72 ± 25.74	58.73 ± 3.598	52.40 ± 12.57	22.15 ± 7.149
**Feces**	41.64 ± 13.96	32.85 ± 5.838	6.733 ± 1.380	**52.64 ± 12.72**	11.21 ± 9.740	2.796 ± 0.435	21.55 ± 8.708

Data are expressed as mean ± SD; *n* = 3. ND: not detected.

**Table 4:** Distribution of resveratrol-3-O-4′-O-disulfate in plasma (nmol/mL), various organs (nmol/g of tissue), urine (nmol/mL), and feces (nmol/g wet content) in mice at different time-points following the intragastric administration of resveratrol at a single dose of 10 mg/kg. Maximum concentrations (C_max_ values) are in bold.

Sample	Resveratrol-3-O-4′-O-Disulfate Concentration						
	0.5 h	1 h	1.5 h	2 h	4 h	6 h	8 h
**Plasma**	**0.099 ± 0.016**	0.090 ± 0.028	0.039 ± 0.023	0.050 ± 0.044	0.033 ± 0.022	0.042 ± 0.037	0.049 ± 0.033
**Spleen**	ND	ND	ND	ND	ND	ND	ND
**Liver**	**0.585 ± 0.258**	0.267 ± 0.143	0.422 ± 0.100	0.517 ± 0.073	0.218 ± 0.036	0.019 ± 0.032	0.021 ± 0.037
**Kidney**	0.430 ± 0.252	0.692 ± 0.516	0.387 ± 0.113	**0.986 ± 0.720**	0.767 ± 0.101	0.247 ± 0.182	0.078 ± 0.046
**Heart**	0.406 ± 0.203	**0.973 ± 0.332**	ND	ND	ND	ND	ND
**Lung**	**0.673 ± 0.867**	0.275 ± 0.177	0.164 ± 0.102	0.438 ± 0.258	ND	ND	ND
**Stomach**	**15.08 ± 9.413**	4.088 ± 3.812	4.899 ± 3.492	1.448 ± 0.834	1.389 ± 0.556	0.251 ± 0.234	0.350 ± 0.076
**Duodenum**	**12.54 ± 6.672**	5.273 ± 4.943	5.080 ± 3.785	1.092 ± 0.104	0.956 ± 0.306	0.203 ± 0.176	0.123 ± 0.113
**Jejunum**	**1.666 ± 1.280**	1.210 ± 0.841	1.233 ± 1.175	0.665 ± 0.191	0.527 ± 0.315	0.166 ± 0.098	ND
**Ileum**	2.264 ± 0.441	2.594 ± 0.577	**2.749 ± 1.006**	1.474 ± 0.938	1.219 ± 0.518	0.260 ± 0.150	0.270 ± 0.168
**Cecum**	0.363 ± 0.319	0.280 ± 0.485	0.807 ± 0.706	**1.123 ± 0.991**	0.305 ± 0.328	ND	ND
**Colon **	0.751 ± 0.287	0.377 ± 0.136	**0.843 ± 0.505**	0.632 ± 0.433	ND	ND	ND
**Thymus**	ND	ND	ND	ND	ND	ND	ND
**Muscle**	0.167 ± 0.089	**0.327 ± 0.167**	0.255 ± 0.214	ND	ND	ND	ND
**Brain**	ND	ND	ND	ND	ND	ND	ND
**Urine**	7.382 ± 0.362	53.17 ± 23.97	10.19 ± 3.056	50.06 ± 24.88	67.57 ± 33.96	**106.0 ± 48.06**	58.26 ± 23.76
**Feces**	**11.92 ± 2.654**	6.930 ± 2.455	1.767 ± 0.060	1.821 ± 0.155	1.989 ± 0.446	7.778 ± 3.473	3.155 ± 5.465

Data are expressed as the mean ± SD; *n* = 3. ND: not detected.

**Table 5:** Area under the concentration–time curves (AUCs) (0–8 h) for plasma, urine, feces, or tissues of resveratrol (Res), resveratrol-3-O-glucuronide (Res3G), resveratrol-3-O-sulfate (Res3S), and resveratrol-3-O-4′-O-disulfate (ResDiS) in mice that received resveratrol (10 mg/kg) intragastrically.

Sample	AUC (nmol/mL min or nmol/g min)						
	Res	Res3G	Res3S	ResDiS	AUC_Res_/AUC_Res3G_	AUC_Res_/AUC_Res3S_	AUC_Res_/AUC_ResDiS_
**Plasma**	1.21 ± 0.72	0.59 ± 0.22	0.71 ± 0.28	0.37 ± 0.15	2.05	1.70	3.27
**Spleen**	27.8 ± 4.35	2.23 ± 1.91	0.28 ± 0.16	n.a.	12.5	99.7	n.a.
**Liver**	2.02 ± 0.44	4.12 ± 2.17	4.59 ± 1.65	1.78 ± 0.56	0.50	0.44	1.13
**Kidney**	4.76 ± 0.88	6.92 ± 2.67	5.56 ± 1.60	3.60 ± 0.99	0.69	0.86	1.32
**Heart**	8.84 ± 3.70	2.00 ± 1.16	1.99 ± 1.41	0.83± 0.37	4.41	4.45	10.6
**Lung**	14.3 ± 3.51	8.27 ± 3.44	6.92 ± 2.88	1.02 ± 0.67	1.73	2.07	14.0
**Stomach**	223 ± 118	84.3 ± 32.7	116 ± 46.2	17.5 ± 5.08	2.65	1.92	12.8
**Duodenum**	17.7 ± 10.1	53.8 ± 26.0	96.9 ± 37.3	15.3 ± 6.54	0.33	0.18	1.16
**Jejunum**	8.33 ± 3.59	18.8 ± 11.3	33.0 ± 16.1	4.27 ± 1.67	0.44	0.25	1.95
**Ileum**	31.1 ± 16.2	92.1 ± 38.6	182 ± 98.2	8.43 ± 3.96	0.34	0.17	0.62
**Cecum**	201 ± 63.5	5.39 ± 3.17	44.9 ± 24.9	2.74 ± 1.12	37.4	4.48	73.5
**Colon**	52.7 ± 22.0	9.87 ± 4.39	14.3 ± 6.12	1.21 ± 0.84	5.34	3.68	43.5
**Thymus**	34.9 ± 12.7	6.19 ± 2.26	14.2 ± 8.35	n.a.	5.63	2.46	0.0
**Muscle**	4.02 ± 1.53	2.37 ± 0.68	0.76 ± 0.21	0.74 ± 0.17	1.70	5.31	5.45
**Brain**	0.46 ± 0.18	n.a.	n.a.	n.a.	n.a.	n.a.	n.a.
**Urine**	38.8 ± 13.6	1521 ± 411	462 ± 103	503 ± 141	0.03	0.08	0.08
**Feces**	1304 ± 337	24.1 ± 12.6	156 ± 52.6	35.3 ± 11.9	54.1	8.36	37.0

Data are expressed as mean ± SD; *n* = 3. n.a.: not applicable.

## Abbreviations

RT-qPCR	reverse transcription-quantitative PCR
Sult	sulfotransferase
Ugt	UDP-glucuronosyltransferase
